# DBALM: A novel method for identifying ornamental flowering plants based on DNA barcodes–leaf morphology

**DOI:** 10.1002/ece3.10250

**Published:** 2023-07-04

**Authors:** Fengjiao Shen, Xiaoxia Bai, Lin Li, Xiaoxuan Fan, Yifan Song, Miao Yu, Mengwei Cui, Shulei Jiang, Zhibin Li, Jiancheng Zhao, Shuo Shi

**Affiliations:** ^1^ College of Life Sciences Hebei Normal University Shijiazhuang China; ^2^ Shijiazhuang Academy of Agriculture and Forestry Sciences Shijiazhuang China; ^3^ Shijiazhuang ShenZhou Flower Insititute Co. Ltd. Shijiazhuang China

**Keywords:** horticulture, leaf epidermis, microscopic characters, molecular markers, vegetative phase

## Abstract

Whereas the presence of flowers on ornamental flowering plants is essential for their identification via traditional methods, ornamental flowering plants cannot be reliably identified in non‐flowering stages likewise. Here, DBALM (**D**NA **Ba**rcodes–**L**eaf **M**orphology), a new approach that combines DNA barcoding data with micromorphological features of the leaf epidermis and that is not limited by the flowering stage, was used to identify 16 evergreen rhododendron cultivars. First, the sequences of DNA barcodes, ITS, *matK*, *psbA‐trnH*, and *rbcL*, were obtained from the DNA of leaves. Phylogenetic analysis was conducted to clarify the groupings among all the samples based on the four markers. Then, microscopic features of the leaf epidermis were used to further distinguish individuals from the same clade. DNA barcoding permitted the 16 cultivars to be divided into eight groups. The microscopic features of the leaf epidermis permitted cultivars within the same clade to be distinguished. The *matK* + *psbA‐trnH* combination was the most effective barcode combination in this study. In addition, the new primer matK‐Rh_R was designed, and it increased the amplification rate of evergreen rhododendron cultivars to 100%. In sum, DBALM was capable of accurately identifying the 16 evergreen rhododendron cultivars using data collected from a single leaf in the vegetative growth stage. This method can greatly facilitate the identification and breeding of ornamental flowering plants.

## Graphical abstract

Whereas the presence of flowers on ornamental flowering plants is essential for identification via traditional identification methods, ornamental flowering plants cannot be reliably identified in non‐flowering stages likewise. Here, DBALM (DNA Barcodes–Leaf Morphology), a new approach that combines DNA barcoding data with micromorphological features of the leaf epidermis and that is not limited by the flowering stage, was used to identify 16 evergreen rhododendron cultivars. This method can greatly facilitate the identification and breeding of ornamental flowering plants.

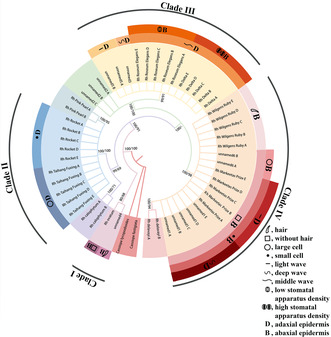

## Abstract (zh)

传统的植物品种鉴定方法主要依据花的形态特征进行对赏花植物进行鉴定。目前，对非花期的赏花植物进行鉴定仍十分困难。为避免花期限制，本研究采用一种新的方法——DNA条形码‐叶形态法(DBALM)对16个高山杜鹃品种进行鉴定，即将DNA条形码与叶表皮微形态特征相结合。首先，利用ITS、*matK*、*psbA‐trnH*和*rbcL*这4个DNA条形码对叶片中的DNA进行扩增及测序，并基于系统发育分析对所有样本进行分组。之后，使用叶表皮显微特征进一步区分系统发育树中同一分支的样品。结果显示，DNA条形码可将16个品种分为8组。叶表皮显微特征可将系统发育树中同一分支内的不同品种进行区分。*matK* + *psbA‐trnH*组合是本研究中最高效的条形码组合。此外，新引物matK‐Rh_R对高山杜鹃品种的扩增率为100%。综上，DBALM可准确对16个高山杜鹃品种进行鉴定。该方法可大大方便赏花植物的品种鉴定和选育。.

## 1 INTRODUCTION

The diversity of economically important ornamental flowering plants is astounding. One of the major challenges in both horticultural research and ornamental plant industry is accurately identifying cultivars. Indeed, the misidentification of cultivars is the cause of major losses to the ornamental plant industry and has major implications for horticultural research. The accurate identification of ornamental flowering plants is thus essential for cultivation and breeding of horticultural plants, including international trade. Although experts in ornamental flowering plants have often been employed for identification, there are several drawbacks associated with this approach (Eagles et al., 2001; Li et al., 2020; Nadeem et al., 2017). First, the identification of ornamental flowering plants via traditional experts requires flowers, but flowering plants may not be accurately identified in non‐flowering stages. Second, plants may be damaged in the process of the collection. For instance, the flowers or branches are likely to be break. Third, there is invariably subjectivity associated with relying on experts for the identification of ornamental flowering plants.

The use of molecular markers and the characterization of morphological traits of vegetative organs can circumvent problems associated with the traditional method of identifying ornamental flowering plants. Molecular marker techniques, in the light of which nucleic acid sequences are used for identification, have been widely used for the identification of various types of plants in recent years (Arif et al., 2010; Azizi et al., 2021; Lu et al., 2019; Sharma et al., 2020; Wang et al., 2018; Yali, 2022). Some other molecular marker techniques are costly, but the amplification rate and stability of the results of these methods are low (Arif et al., 2010; Azizi et al., 2021; Nadeem et al., 2017; Shi et al., 2016; Yali, 2022). DNA barcoding has been widely used for classification and identification of plants because of its simplicity and high repeatability (Liu et al., 2021; Vere et al., 2015). However, the resolution of DNA barcoding is not sufficiently high to permit closely related cultivars to be distinguished, as cultivars vary in ploidy and bud mutations (Azizi et al., 2021; Shi et al., 2016; Yan et al., 2015). Morphological features of vegetative organs, such as micromorphology of the leaf epidermis, ultrastructural features, and structure of leaf veins, have frequently been used to classify and identify plants (Cristina et al., 2008; Franklin, 1945; Keating, 1984; Payne, 1979; Silva et al., 2016; Wang et al., 2006). Observations of micromorphological features of the leaf epidermis can be easily made. The micromorphological features of bud mutants and polyploid cultivars differ from those of stock plants (Chen et al., 2009, 2010; Yi et al., 2016). However, the micromorphological features that are currently used for the identification of cultivars is inefficiency for classifying numerous cultivars. There is, thus, an urgent need to establish an affordable and efficient method for the identification of ornamental flowering plants, which does not require identification expertise. In this study, DNA barcoding and observations of micromorphological features of the leaf epidermis, DNA Barcodes–Leaf Morphology (DBALM), were used to circumvent some of the various shortcomings of traditional identification methods.

We used cultivars of evergreen rhododendron plants to evaluate the efficacy of DBALM. Evergreen rhododendron cultivars are members of the genus *Rhododendron* with large, evergreen leaves and racemes. Thousands of cultivars in the genus *Rhododendron* are registered in the Royal Horticultural Society (Bai et al., 2019; The Royal Horticultural Society, 2022). Evergreen rhododendron cultivars are members of a large group of ornamental flowering plants in the genus *Rhododendron*. Given that most of these cultivars are derived from hybridization, bud mutations, and polyploidy, robust methods are needed that permit identification of evergreen rhododendron cultivars.

In this research, we used 16 evergreen rhododendron cultivars to evaluate the efficacy of DBALM for the identification of ornamental flowering plants. The ultimate aim of this study was to develop a convenient, low‐cost and extensively applicable method for the identification of ornamental flowering plants when they are at vegetative growth stage.

## 2 MATERIALS AND METHODS

### 2.1 Materials

A total of 16 evergreen rhododendron cultivars, two cultivars in subg. *Azaleastrum* and 14 cultivars in subg. *Hymenanthes*, were used to test the efficacy of DBALM in this study. DNA barcoding was conducted on samples taken from 51 individual plants. Data on the micromorphological features of the mature leaves were collected from 25 individual plants. Information on the experimental materials is shown in Table 1. *Cassiope fastigiata* and *Cassiope lycopodioides* were used as the outgroups per Khan et al. (2021) (Table 2). *Rh*. “Roseum Elegans” and *Rh*. “Delta” were used to evaluate the consistency of the micromorphological features of young and mature leaves in evergreen rhododendron cultivars. The voucher specimens were deposited in the herbarium of Hebei Normal University, China (HBNU).

**TABLE 1:** Information on the experimental materials used for the collection of DNA barcoding data and micromorphological data of the leaf epidermis of Rhododendron.

No.	Subgenus	Names	DNA barcoding		Leaf epidermis microscopic characteristics		Collectors
			Number of individuals	Collection numbers	Number of individuals	Specimens for leaf epidermis	
1	*Hymenanthes*	*Rh*. “Taihang Fuxing”	5	R01–R05	2	R01/R03	Shen et al.
2	*Hymenanthes*	UNNAMED‐1	5	R06–R10	2	R06/R07	Shen et al.
3	*Hymenanthes*	*Rh. “*Roseum Elegans”	5	R11–R15	2	R11/R12(Y)	Shen et al.
4	*Hymenanthes*	*Rh. “*Wilgens Ruby”	5	R16–R20	2	R16/R17	Shen et al.
5	*Hymenanthes*	*Rh. “*Markeeta's Prize”	5	R21–R25	2	R21/R23	Shen et al.
6	*Hymenanthes*	*Rh*. “Delta”	5	R26–R30	2	R26/R29(Y)	Shen et al.
7	*Hymenanthes*	*Rh*. “Rocket”	5	R31–R35	2	R31/R35	Shen et al.
8	*Hymenanthes*	UNNAMED‐2	3	R41/R43/R44	2	R41/R44	Shen et al.
9	*Hymenanthes*	UNNAMED‐3	1	R42	1	R42	Shen et al.
10	*Azaleastrum*	UNNAMED‐4	1	R46	1	R46	Shen et al.
11	*Hymenanthes*	*Rh. calophytum*	2	R51/R52	2	R51/R52	Shen et al.
12	*Azaleastrum*	*Rh. ovatum*	1	R56	1	R56	Shen et al.
13	*Hymenanthes*	UNNAMED‐5	2	R61–R62	1	R62	Shen et al.
14	*Hymenanthes*	*Rh. delavayi*	2	R66–R67	1	R67	Bai et al.
15	*Hymenanthes*	*Rh*. “Pink Pearl”	2	R71–R72	1	R72	Bai et al.
16	*Hymenanthes*	UNNAMED‐6	2	R76–R77	1	R77	Bai et al.
Total			51		25		

*Note*: (Y), young leaves.

**TABLE 2:** Accession numbers of the outgroups in GenBank for DNA barcoding.

No.	Subfamilies	Scientific names	Accessions No. in GenBank			
			ITS	*matK*	*psbA‐trnH*	*rbcL*
O1	Andromededoideae	*Cassiope fastigiata*	KR819516.1	JF953430.1	JN044178.1	KR819573.1
O2	Andromededoideae	*Cassiope lycopodioides*	HM182074.1	AB012754.1	–	KX678866.1

### 2.2 Methods

#### 2.2.1 DNA barcoding and micromorphological features of the leaf epidermis

DNA barcoding data can provide insights into the genetic relationships among cultivars and aid the breeding and domestication of cultivars, as well. DNA barcoding requires fewer materials and can be conducted more rapidly in batches than observations of the micromorphology of the leaf epidermis. Thus, we used DNA barcoding to preliminarily classify the 16 evergreen rhododendron cultivars, before we used micromorphological features of the leaf epidermis to facilitate identification.

#### 2.2.2 Identification based on DNA barcodes

Molecular samples from fresh material were obtained via the drying treatment method at 40°C to minimize the degradation of DNA (Shen et al., 2017, 2022). The mCTAB method was used to extract total DNA (Li et al., 2013). Four markers used in previous studies of members of the genus *Rhododendron* (Fu et al., 2022; Liu, 2011; Liu et al., 2012; Ma et al., 2013; Yan et al., 2015), ITS, *rbcL*, *matK*, and *psbA‐trnH*, were used in our DNA barcoding analysis. Primers and references for the PCR protocol are provided in Table 3. The amplification success rate of *matK* was low (Liu, 2011; Liu et al., 2012; Ma et al., 2013; Yan et al., 2015). We could not obtain a single positive result when the primers from previous studies were used for the amplification. Thus, we designed a new primer, matK‐Rh_R (5′‐GCCCGCTATAATAATGAGAAAGAYTTC‐3′), from the *matK* sequence in GenBank.

**TABLE 3:** Primers for the four molecular markers of DNA barcoding.

Markers	Primer names	Primer sequences (5′–3′)	References
ITS	ITS‐P5	CCTTATCAYTTAGAGGAAGGAG (22)	Cheng et al. (2016)
	ITS‐U4	RGTTTCTTTTCCTCCGCTTA (20)	
*rbcL*	rbcLb‐sF	AGACCTTTTTGAAGAAGGTTCTGT (24)	Dong et al. (2014)
	rbcLb‐sR	TCGGTCAGAGCAGGCATATGCCA (23)	
*matK*	matK‐472F	CCCRTYCATCTGGAAATCTTGGTTC (25)	Yu et al. (2011)
	matK‐1248R	GCTRTRATAATGAGAAAGATTTCTGC (26)	
	matK‐Rh_R	GCCCGCTATAATAATGAGAAAGAYTTC (27)	In this study
*psbA‐trnH*	psbA‐3(F)	GTTATGCATGAACGTAATGCTC (22)	Sang et al. (1997)
	trnH‐3(R)	CGCGCATGGTGGATTCACAATCC (23)	

MAFFT v7.130b (Katoh & Standley, 2013) was used to align all sequences prior to phylogenetic analysis while Sequencher v5.4 (Gene Codes Corporation) was used to perform quality control. Phylogenetic trees were constructed with all the samples via the Bayesian inference (BI) and maximum likelihood (ML) methods in Phylosuit v1.1.14 (Zhang et al., 2020) via MrBayes v3.2.1 (Ronquist et al., 2012) and IQ‐TREE (Nguyen et al., 2015), respectively. We conducted two independent simultaneous runs (three hot chains and one cold chain for each run), and sampling was conducted every 1000 generations for 1,000,000 generations. The average standard deviation of the split frequencies was less than 0.01. ML analysis was conducted by using 10,000 ultrafast bootstraps (Minh et al., 2013). The BI and ML models (Kalyaanamoorthy et al., 2017; Lanfear et al., 2017) are shown in Table A1 in Appendix 1. The tree was plotted via using iTOL (https://itol.embl.de/).

#### 2.2.3 Micromorphological trait‐based identification

**FIGURE 1:**
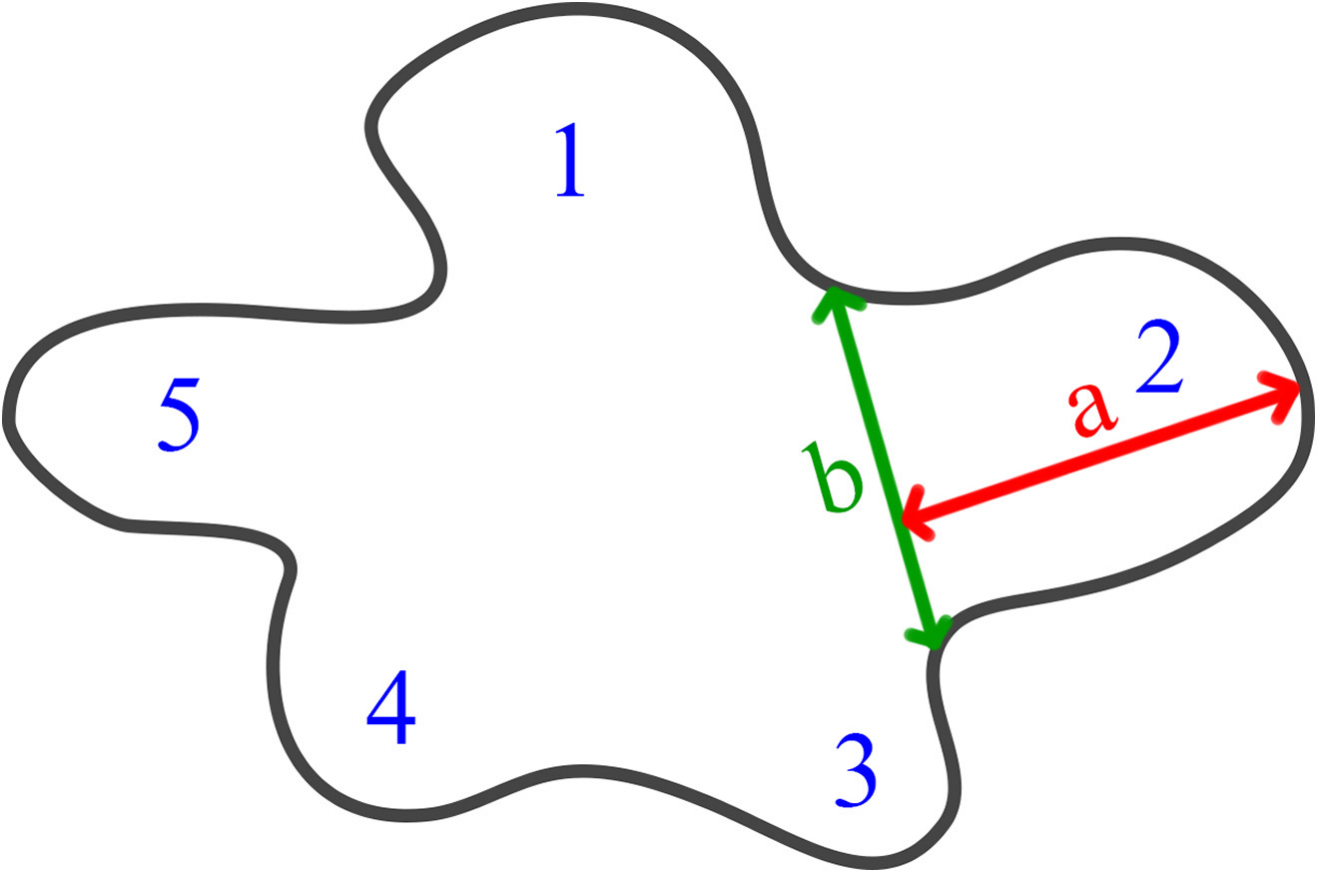
Schematic diagram of the height and width of the anticlinal wall waves of an epidermal cell. *Note*: a, the width of the wave of the cell; b, the height of the wave of the cell; 1–5, the waves of the cell.

Water‐mounted slides of leaf epidermis were prepared following the method of Wang et al. (2007). Four 1 × 1‐cm pieces of the leaves of each individual were collected and used to make micromorphological observations. A Nikon E800 optical microscope, NIS‐Elements F 3.0 software, and ImageJ v1.8.0 software (http://rsbweb.nih.gov/ij/) were used to characterize the size of cells, anticlinal wall pattern, height and width of anticlinal wall waves, stomata density and the presence of hairs. In this study, a measurement (Figure 1) and data analysis was conducted on the height and width ratio of the anticlinal wall waves. Variation in character among cultivars is intermittent when there is no overlap in the boxplots, and the character can be used to distinguish cultivars. The terminology of the micromorphological traits of the leaf epidermis followed that of Dilcher (1974). SPSS v23.0 was used to conduct statistical analyses.

## 3 RESULTS

### 3.1 DNA barcode‐based identification

Table 4 shows the lengths of the markers and the success rates of PCR amplification and sequencing. The success rate of PCR amplification was 100% for all the markers. The success rate of sequencing was 100% for all the chloroplast makers and 98.04% for the ITS sequences. All of sequences have been uploaded to GenBank (Table A2 in Appendix 2).

**TABLE 4:** PCR results of the different DNA barcodes.

	ITS	*matK*	*psbA‐trnH*	*rbcL*
Success rates of PCR (%)	100	100	100	100
Success rates of sequencing (%)	98.04	100	100	100
Sequence lengths (bps)	646–698	470–793	263–468	410–757

**FIGURE 2:**
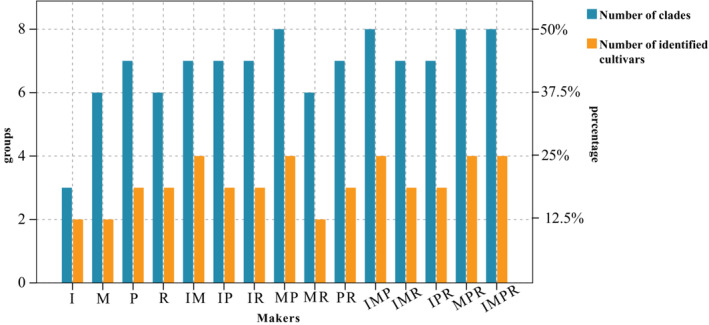
Number of groups of the 16 evergreen rhododendron cultivars in trees built using different DNA barcodes. *Note*: I, ITS; M, *matK*; P, *psbA‐trnH*; R, *rbcL*.

**FIGURE 3:**
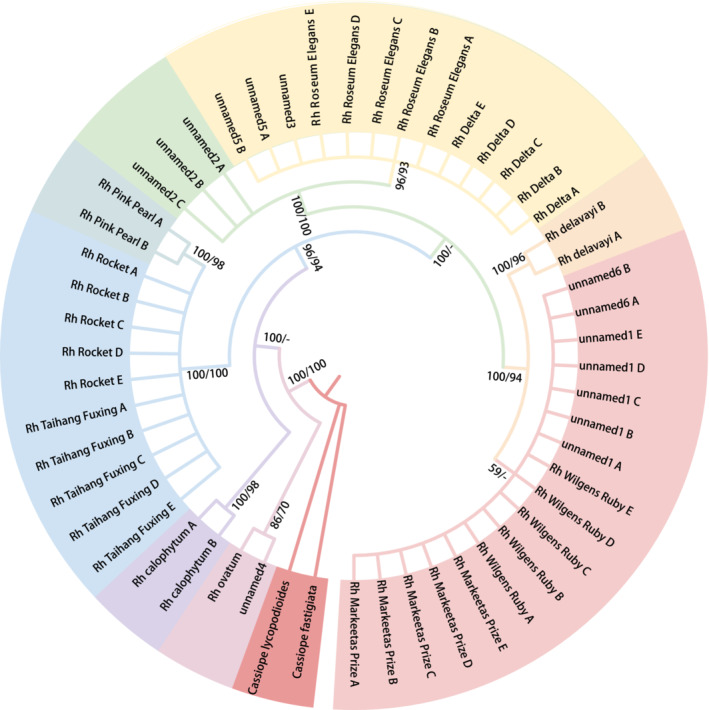
Phylogenetic tree built via the Bayesian inference and maximum likelihood methods using the ITS + *matK* + *psbA‐trnH* + *rbcL* combination of markers. *Note*: Bayesian inference (BI) and ML bootstrap supports are shown at the nodes (BI/ML).

**FIGURE A1:**
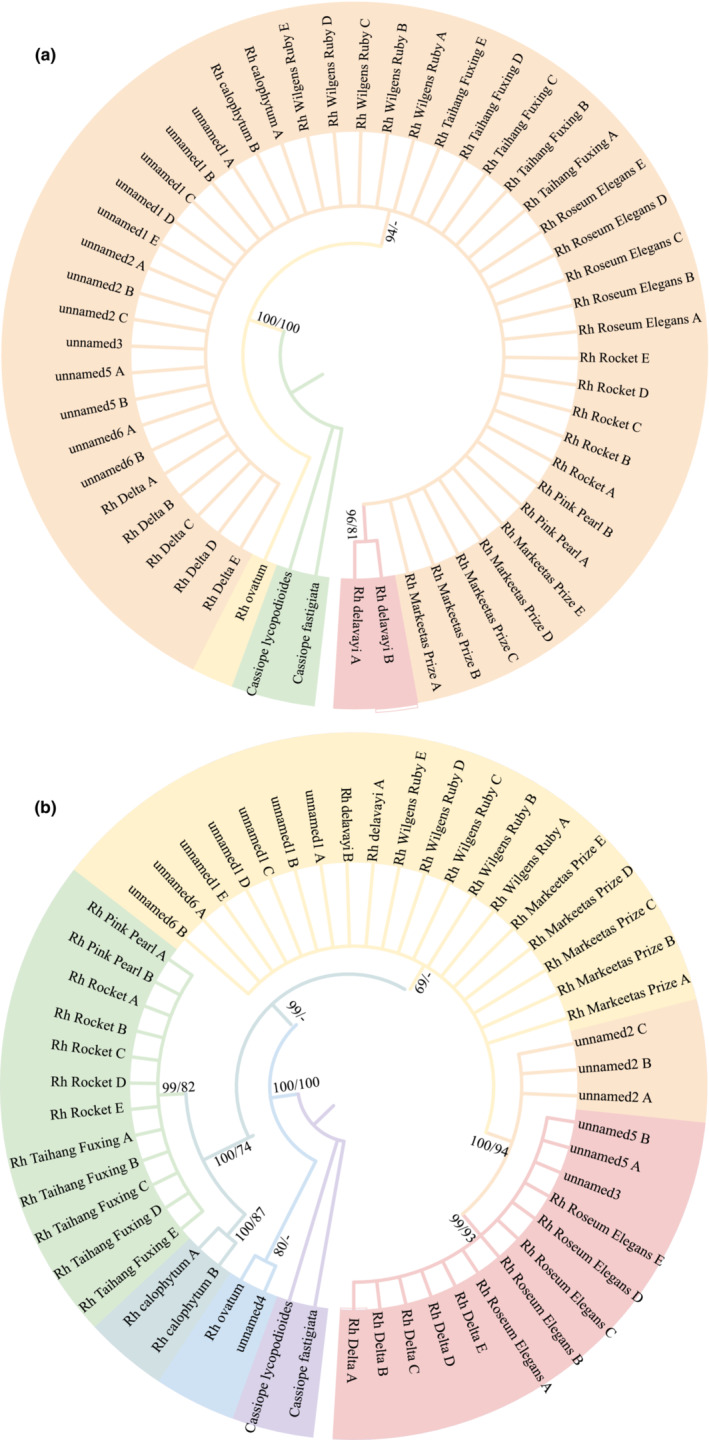
Phylogenetic trees built via the BI and ML methods using the sequences of four makers. (a) ITS; (b) *matK*.

**FIGURE A2:**
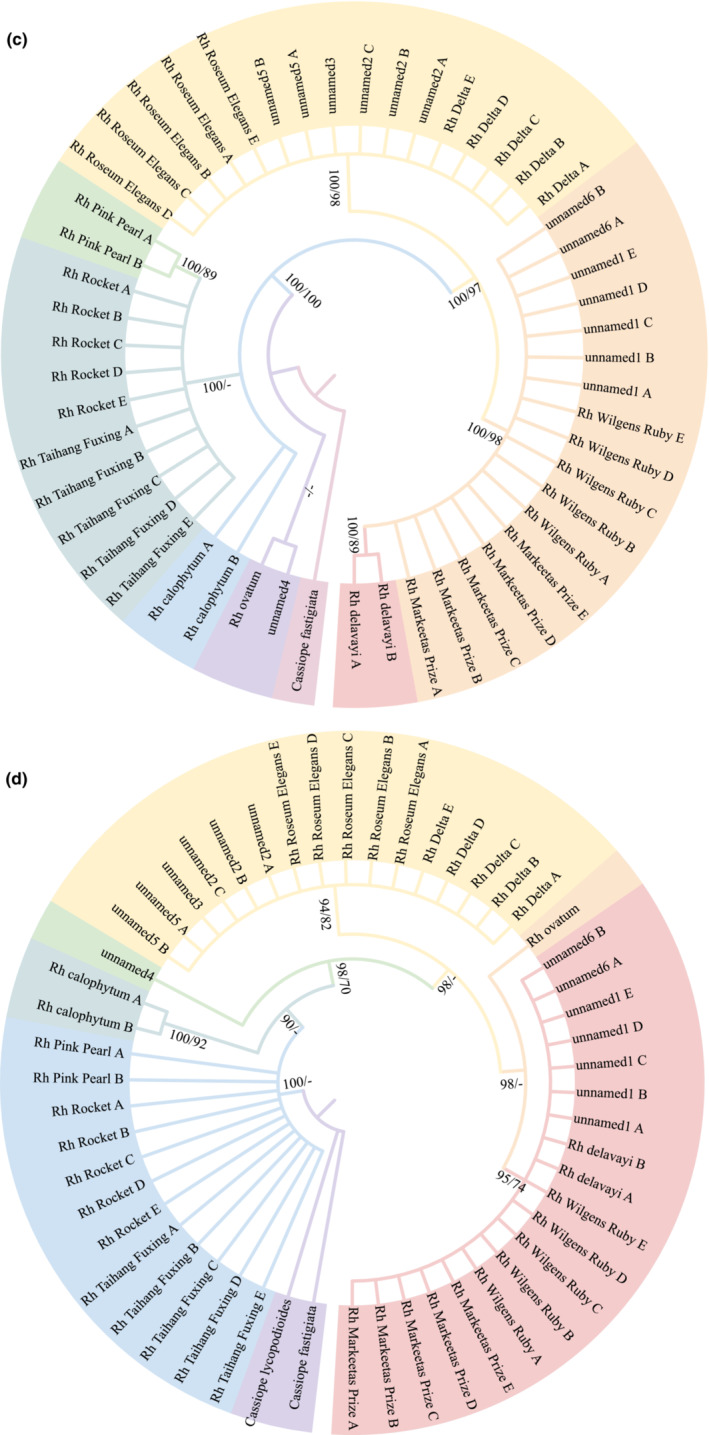
Phylogenetic trees built via the BI and ML methods using the sequences of four makers. (c) *psbA‐trnH*; (d) *rbcL*.

**FIGURE A3:**
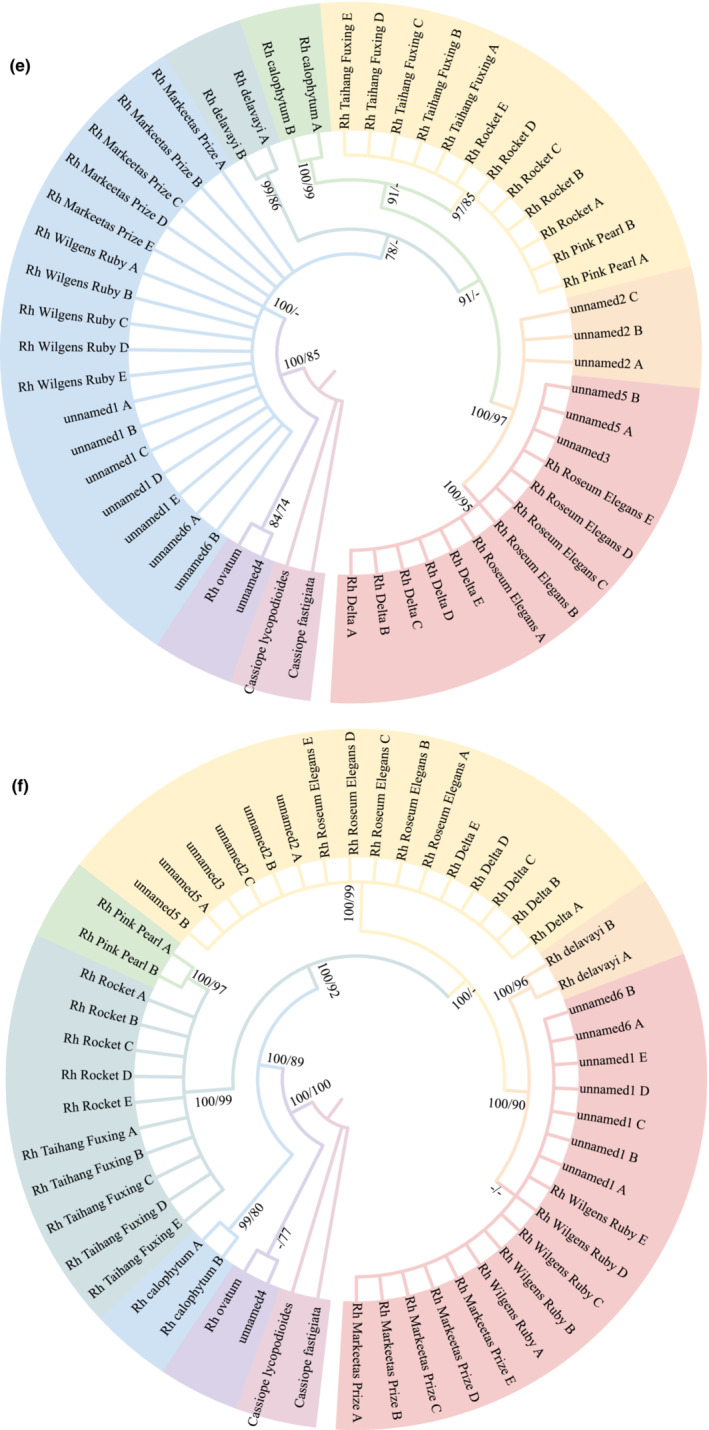
Phylogenetic trees built via the BI and ML methods using the sequences of four makers. (e) ITS + *matK*; (f) ITS + *psbA‐trnH*.

**FIGURE A4:**
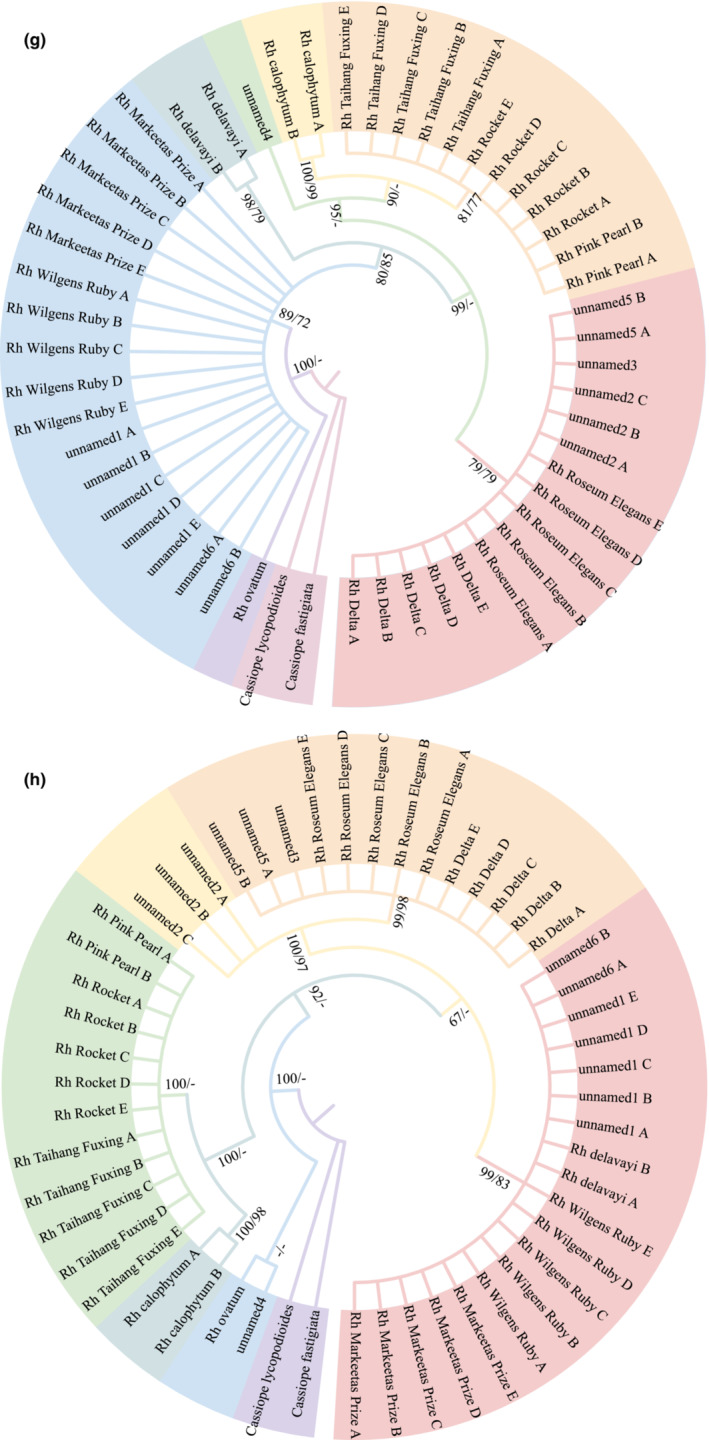
Phylogenetic trees built via the BI and ML methods using the sequences of four makers. (g) ITS + *rbcL*; (h) *matK* + *rbcL*.

**FIGURE A5:**
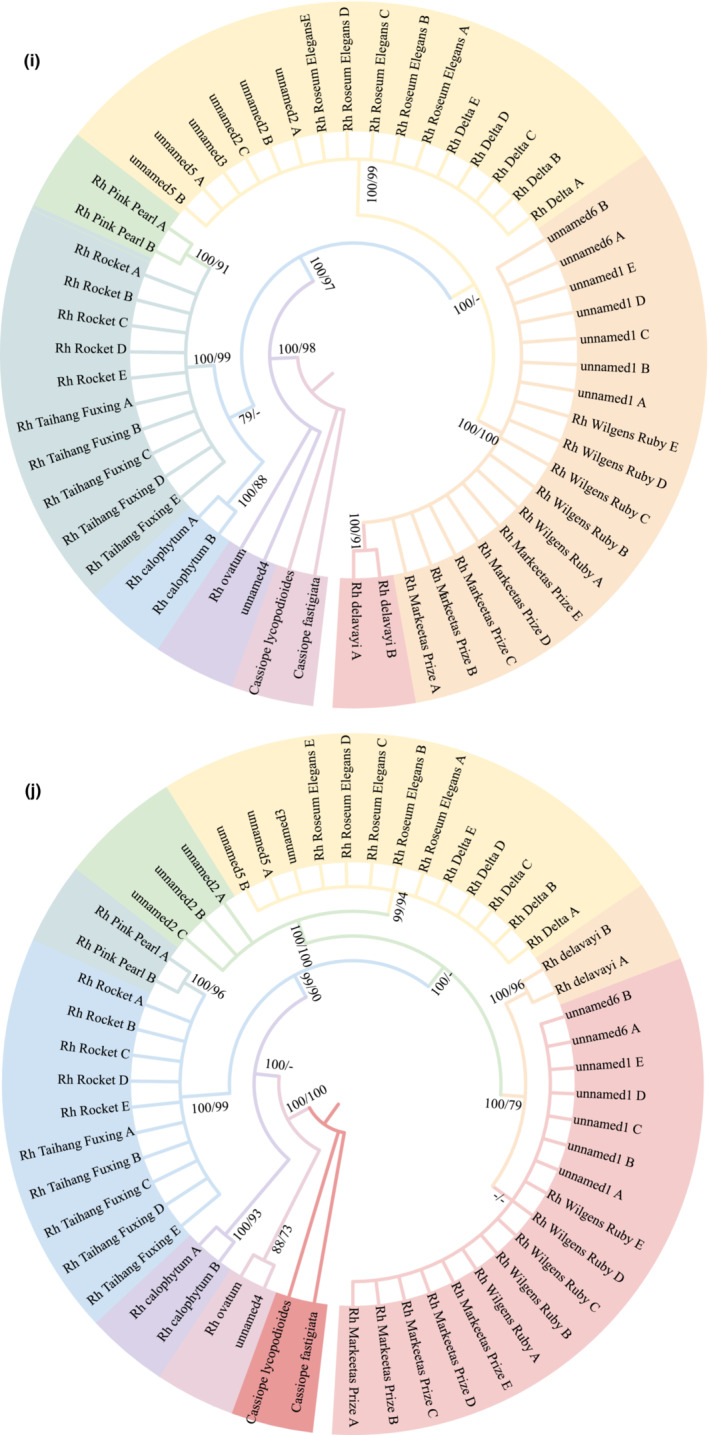
Phylogenetic trees built via the BI and ML methods using the sequences of four makers. (i) *psbA‐trnH* + *rbcL*; (j) ITS + *matK* + *psbA‐trnH*.

**FIGURE A6:**
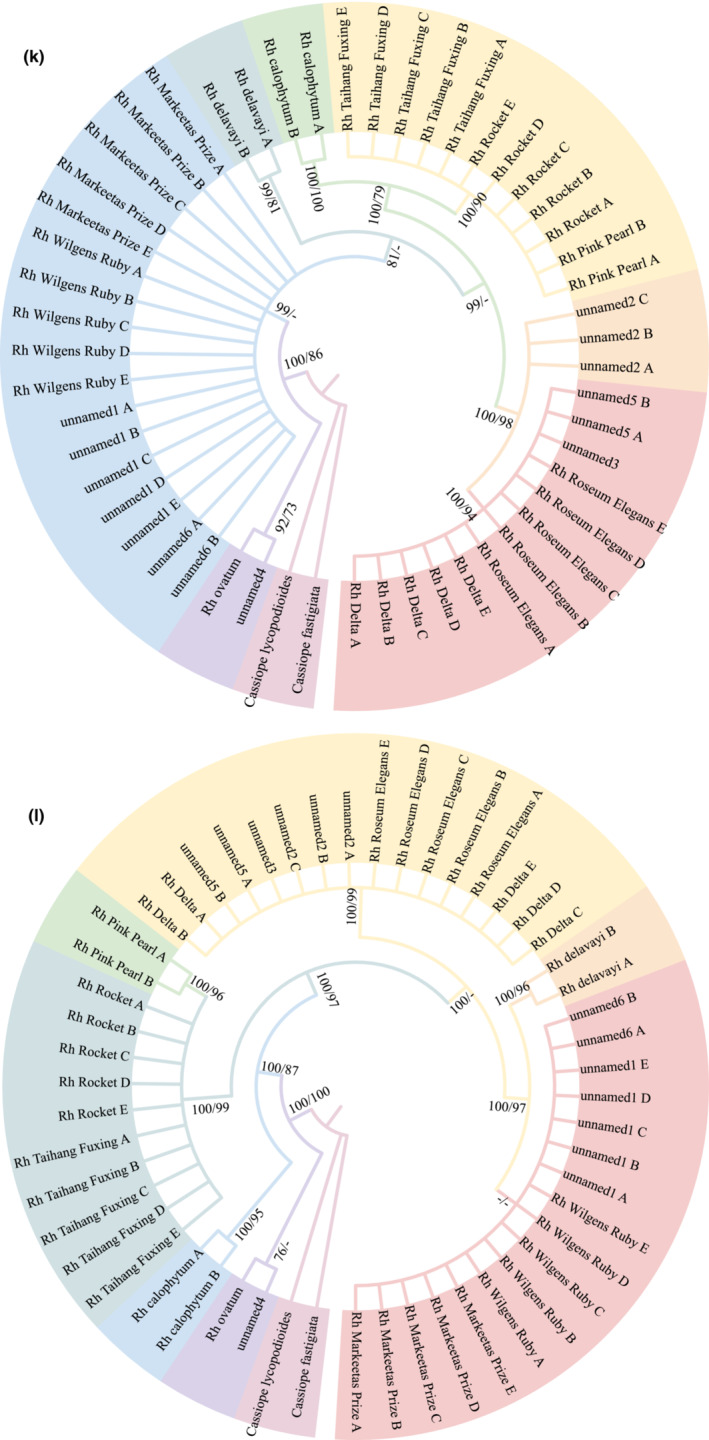
Phylogenetic trees built via the BI and ML methods using the sequences of four makers. (k) ITS + *matK* + *rbcL*; (l) ITS + *psbA‐trnH* + *rbcL*.

**FIGURE A7:**
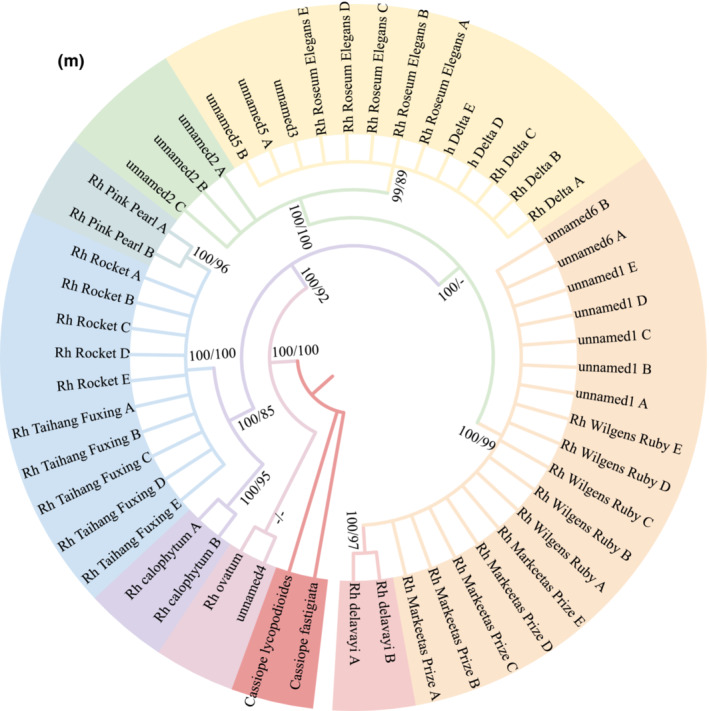
Phylogenetic trees built via the BI and ML methods using the sequences of four makers. (m) *matK* + *psbA‐trnH* + *rbcL*.

Phylogenetic trees were constructed using the matrices with both single markers and multiple markers (Figures A1, A2, A3, A4, A5, A6, A7 in Appendix 3). In the trees, the samples were divided into several clades and clustered by cultivar via DNA barcoding. The number of clades was showed in Figure 2. The resolution provided by the primers *psbA‐trnH* was highest when single genes were used to construct phylogenetic trees of evergreen rhododendron cultivars. No differences were observed in the resolution provided by *psbA‐trnH* + *matK* or three‐gene or four‐gene combinations, while the topologies of all trees suggested that the 16 evergreen rhododendron cultivars could be divided into eight clades (Figures 2 and 3). Only 4 of the 16 cultivars (25%) could be distinguished at most. The remaining 12 cultivars were divided into four clades, each comprising more than one cultivar. The two cultivars UNNAMED‐4 and *Rh*. “ovatum” comprised clade I. The two cultivars *Rh*. “Taihang Fuxing” and *Rh*. “Rocket” comprised clade II. The four cultivars UNNAMED‐5, UNNAMED‐3, *Rh*. “Roseum Elegans,” and *Rh*. “Delta” comprised clade III, and the four cultivars UNNAMED‐6, UNNAMED‐1, *Rh*. “Wilgens Ruby,” and *Rh*. “Markeetas Prize” comprised clade IV.

### 3.2 Efficacy of DBALM

**FIGURE A8:**
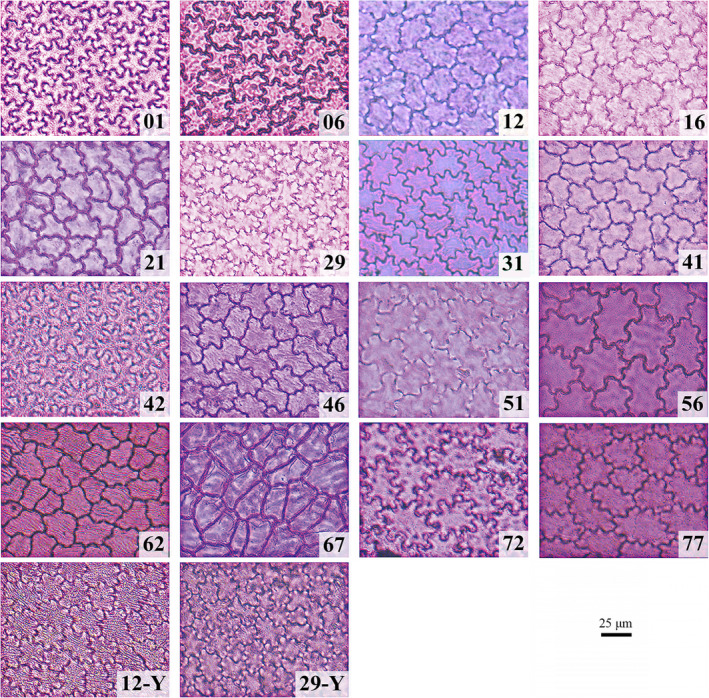
Adaxial epidermal micromorphological characteristics of evergreen rhododendron cultivars. *Note*: The lower right corner of the small figure represents the specimen collection numbers.

**FIGURE A9:**
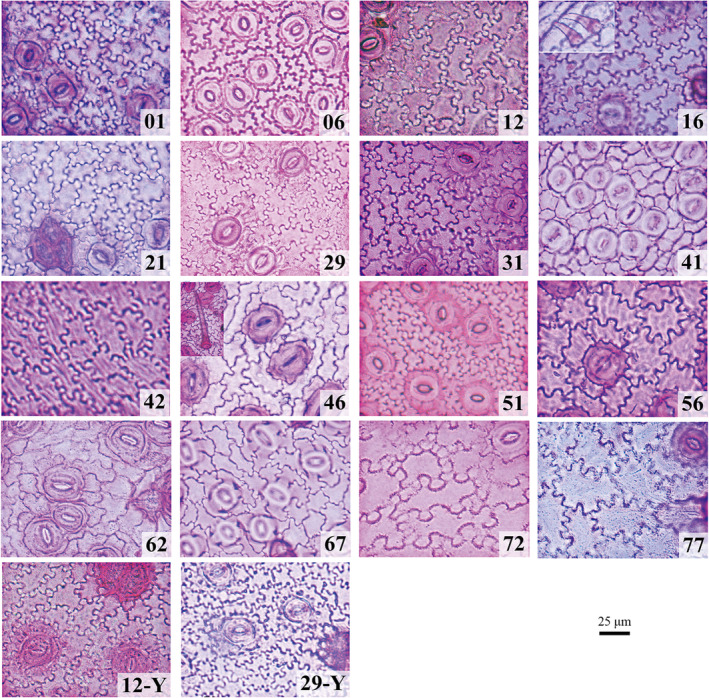
Abaxial epidermal micromorphological characteristics of evergreen rhododendron cultivars. *Note*: The lower right corner of the small figure represents the specimen collection numbers (in Table 1).

The clades generated via the DNA barcoding data were divided into smaller subclades according to micromorphological features of the leaf epidermis. The materials used for the collection of the micromorphological data from the leaf epidermis are shown in Figures A8 and A9 in Appendices 4 and 5. Cell size, anticlinal wall pattern, stomata density, and the hair on the abaxial epidermis permitted the 12 cultivars in clades I, II, III, and IV to be distinguished. The length and width of the anticlinal wall waves of 80 adaxial epidermal cells in different locations in each sample of the six cultivars were measured. A total of 4647 height and width measurements were taken, and the ratios of the height and width measurements were calculated. The micromorphological features of the leaf epidermis are consistent in terms of key morphological features among individual evergreen rhododendron plants within the same cultivar or among individuals in different developmental stages.

**FIGURE 4:**
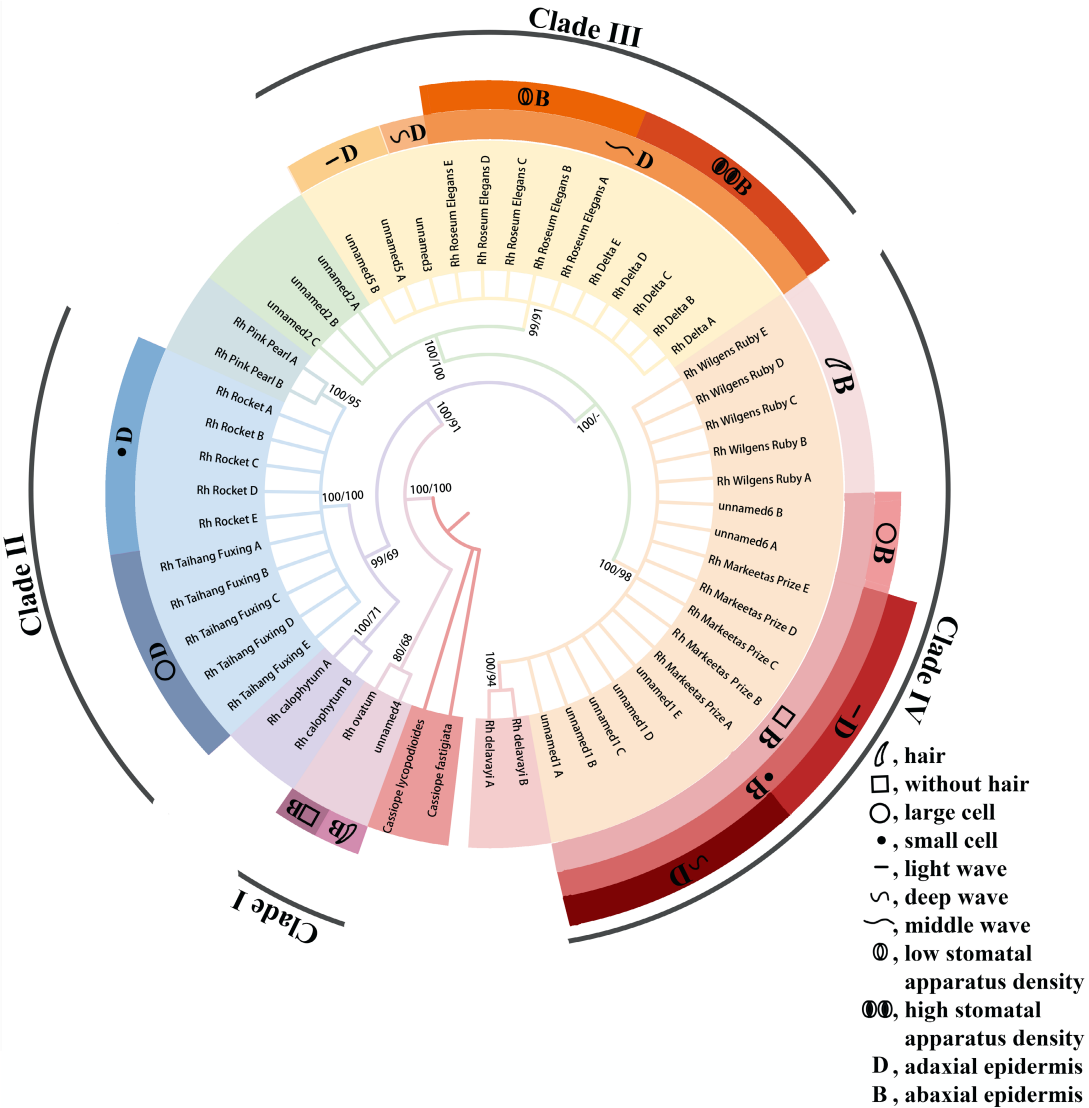
Phylogenetic tree built via the Bayesian inference (BI) and maximum likelihood (ML) methods using the *matK* + *psbA‐trnH* combination of markers. *Note*: Bayesian inference (BI) and ML bootstrap supports are shown at the nodes (BI/ML). The groups are indicated in different colors. The inner rings with cultivar names indicate the clades identified using DNA barcoding; the outer rings with letters indicate the groups identified using leaf epidermal micromorphological characteristics.

**FIGURE 5:**
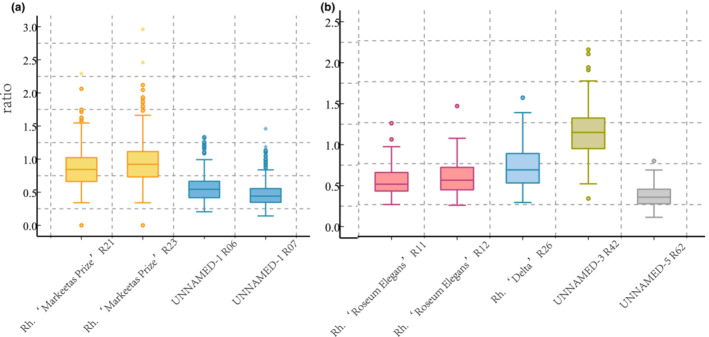
Height‐to‐width ratio of adaxial epidermal cell waves in the leaves of six evergreen rhododendron cultivars. Cultivar names and collection numbers are marked on the *X*‐axis. *Note*: (a) the ratio of Rh. “Markeetas Prize” and UNNAMED‐1; (b) the ratio of Rh. “Roseum Elegans”, Rh. “Delta”, UNNAMED‐3 and UNNAMED‐5.

In clade I (Figure 4), some hairs were present in the abaxial epidermis of UNNAMED‐4, and hairs were lacking in the abaxial epidermis of *Rh*. “ovatum.” In clade II, the adaxial epidermal cells of *Rh*. “Taihang Fuxing” were small (approximately 350 μm^2^ for each cell), and the adaxial epidermal cells of *Rh*. “Rocket” were large (approximately 550 μm^2^ for each cell). Clade III contained four cultivars, UNNAMED‐3, *Rh*. “Roseum Elegans,” *Rh*. “Delta,” and UNNAMED‐5. The waves of the adaxial epidermal cells were deepest in UNNAMED‐3 (Figure 5b). UNNAMED‐5 had the shallowest waves in the adaxial epidermal cells (Figure 5b). The stomatal apparatus density of *Rh*. “Delta” (about 380 stomatal apparatus per square millimeter) were denser than those of *Rh*. “Roseum Elegans” (about 240 stomatal apparatus per square millimeter). *Rh*. “Wilgens Ruby” was the only cultivar with hair in the abaxial epidermis in clade IV. In the remaining three glabrous cultivars of clade IV, the abaxial epidermal cells of UNNAMED‐6 were large (1000 μm^2^), and *Rh*. “Markeetas Prize” and those of UNNAMED‐1 were small (500 μm^2^). The waves of the cell wall of the epidermis in adaxial cells were deeper in UNNAMED‐1 than in *Rh*. “Markeetas Prize” (Figure 5a).

DNA barcoding (*matK* + *psbA‐trnH*), coupled with analysis of microscopic features of the leaf epidermis, revealed that the 16 evergreen rhododendron cultivars could be divided into 16 groups. Thus, the accuracy rate of DBALM for the distinguish of evergreen rhododendron plants was 100%.

## 4 DISCUSSION

In this study, a combination of DNA barcoding and leaf epidermis microscopic characteristics (DBALM) was used to distinguish 16 evergreen rhododendrons. The aim of this study is to find a new method for the identification of ornamental flowering plants without restriction of growth stage. The results showed that DBALM could distinguish 16 evergreen rhododendron and could be used for the identification of horticultural cultivated plants. DNA barcoding data can provide insights into the genetic relationships among cultivars and aid their breeding and domestication. DNA barcoding can be used to classify the cultivars into different groups. Cultivars in the same group are genetically related. Micromorphological features of the leaf epidermis can then be used to classify the cultivars into different subgroups. The resolution provided by the *matK* + *psbA‐trnH* combination for the identification of evergreen rhododendron cultivars was the same as that provided by the ITS + *matK* + *psbA‐trnH* + *rbcL* combination. The highest resolution for the identification of the evergreen rhododendron cultivars is provided by the four‐marker combination, according to previous studies (Yan et al., 2015). Thus, the *matK* + *psbA‐trnH* combination is sufficient for distinguishing between evergreen rhododendron cultivars via DNA barcoding.

Recent studies (Fu et al., 2022) have shown that DNA barcoding has a 35% success rate for identifying wild species in the genus *Rhododendron*. But when the chloroplast genome combined with nuclear genes was used for identification, success rate was increased to 55%. In contrast, the identification of cultivars of evergreen rhododendrons might be more complicated than that of wild species because of the close relationships among cultivars. In fact, success rate of the identification of the cultivars of evergreen rhododendrons using the same DNA barcodes (ITS + *matK* + *psbA‐trnH* + *rbcL*) in this study was 10% lower than that of wild species in the genus *Rhododendron* (Fu et al., 2022). However, when DBALM was used to identify the cultivars of evergreen rhododendrons, success rate was 100%. Therefore, we speculate that DBALM has a higher success rate than the use of chloroplast genomes and nuclear genes for the identification of cultivars in the genus *Rhododendron* and that it has broad application prospects. DBALM enhanced the efficiency and accuracy of identification of evergreen rhododendron cultivars.

DNA barcoding and leaf epidermis microscopic characteristics are widely used in the classification of horticultural cultivars, such as banana and orange (Yi et al., 2016; Zulkifli, 2013). Therefore, although this study only used 16 evergreen rhododendrons as an example, we speculate that this new method could be effective for the identification of a variety of cultivated evergreen plants. The advantages of DBALM are manifold. First, cultivars can be identified at any growth stage, including non‐flowering stages. Second, few materials are needed for identification using this method that minimizes damage to the plant. Third, cultivars can be performed by those lacking expertise in particular plant groups. Fourth, the results of the identification procedure are repeatable and objective. Fifth, the procedure can be performed relatively rapidly and is less costly. However, the accuracy of DBALM identification is highly dependent on the comprehensiveness and accuracy of the database. Therefore, on the one hand, DNA barcode screening and database improvement of horticultural plants should be strengthened, and on the other hand, the construction of leaf epidermis characteristic database of horticultural plants should be strengthened. In addition, manual statistics of the vertical wall patterns of leaf epidermal cells is time‐consuming and laborious. Therefore, AI images recognition technology can be introduced to improve work efficiency and accuracy in the future mass production of leaf epidermal microscopic feature database. The advantage of micromorphological characters used in plant identification is the amount of materials is small, the identification time is short, the experimental operation is easy, not restricted by the growth stage. And the disadvantages are the limit of database of the leave character of different taxa, and some leaf features are not readily available, such as the veins of leathery leaves. Chances are that the efficacy of our method could be improved through the development of a germplasm resource bank of cultivars, as a germplasm resource database that integrates DNA barcode sequences and micromorphological features of the leaf epidermis could be used to generate a unique label for each cultivar.

DBALM can also be used for the identification of deciduous plants and wild plants, including taxonomically complex groups. Although genomics is currently a heated topic in the fields of identification, taxonomy, phylogeny, evolution, and biodiversity conservation, it might not be the most effective approach in many cases, especially for cultivar identification. The potential value of morphological characteristics, including micromorphological characteristics, should not be ignored.

## 5 CONCLUSION

DBALM permitted accurate identification of 16 evergreen rhododendron cultivars by data collected from a single leaf in the vegetative growth stage. This method could greatly facilitate the identification and breeding of rhododendron cultivars and other ornamental cultivars.

## AUTHOR CONTRIBUTIONS

**Fengjiao Shen:** Conceptualization (equal); data curation (equal); formal analysis (equal); funding acquisition (equal); investigation (equal); methodology (equal); software (equal); validation (equal); writing – original draft (equal); writing – review and editing (equal). **Xiaoxia Bai:** Funding acquisition (equal); investigation (equal); project administration (equal); resources (equal); writing – review and editing (equal). **Lin Li:** Conceptualization (equal); data curation (equal); formal analysis (equal); funding acquisition (equal); methodology (equal); writing – original draft (equal); writing – review and editing (equal). **Xiaoxuan Fan:** Data curation (equal); software (equal); validation (equal); visualization (equal). **Yifan Song:** Validation (equal); visualization (equal); writing – original draft (equal). **Miao Yu:** Validation (equal); visualization (equal). **Mengwei Cui:** Validation (equal); visualization (equal). **Shulei Jiang:** Data curation (equal); resources (equal); supervision (equal). **Zhibin Li:** Funding acquisition (equal); project administration (equal); resources (equal). **Jiancheng Zhao:** Funding acquisition (equal); methodology (equal); resources (equal); writing – original draft (equal); writing – review and editing (equal). **Shuo Shi:** Conceptualization (equal); data curation (equal); formal analysis (equal); investigation (equal); methodology (equal); project administration (equal); resources (equal); software (equal); supervision (equal); validation (equal); visualization (equal); writing – original draft (equal); writing – review and editing (equal).

## FUNDING INFORMATION

This work was supported by the (Natural Science Foundation of Hebei Province) under Grant (number C2019205175), (Innovation Fund Project for Graduate Students of Hebei Province) under Grant (number CXZZBS2019088), (Modern Flower Germplasm Innovation Team of Hebei Province) under Grant (number 21326317D), and (International Cooperation Project of Shijiazhuang Science and Technology Project 2022) under Grant (number 221490034A).

## Data Availability

The alignments of the ITS, *matK*, *psbA‐trnH*, and *rbcL* sequences of 16 cultivars evergreen rhododendrons are deposited in Science DB (https://doi.org/10.57760/sciencedb.06685); Height‐to‐width ratios of adaxial epidermal cell waves of six cultivars evergreen rhododendrons in Figure 5 are deposited in Science DB (https://doi.org/10.57760/sciencedb.06720). The sequencing data are available in GenBank. GenBank accession numbers are in Table A2 in Appendix 2.

## APPENDIX 1

**TABLE A1:** Models used for all single and multiple genes for phylogenetic tree.

	BI	ML
ITS	K80 + G	K80 + I
*matK*	F81 + F	K81u + F
*psbA‐trnH*	HKY + G + F	K81u + G4 + F
*rbcL*	JC + I	JC + I
ITS + *matK*	K2P + I/F81 + F	K2P + I/K3Pu + F + G4
ITS + *psbA‐trnH*	K2P + I/F81 + F + G4	K2P + G4/K3Pu + F + G4
ITS + *rbcL*	K2P + I/K2P + I	K2P + G4/JC + I
*matK* + *psbA‐trnH*	F81 + F/HKY + F + G4	K3Pu + F/K3Pu + F + G4
(*matK* + *rbcL*)	(F81 + F/JC + I)	(K3Pu + F + I)
*psbA‐trnH* + *rbcL*	HKY + F + G4/JC + I	K3Pu + F + G4/JC + I
ITS + *matK* + *psbA‐trnH*	K2P + G4/F81 + F/F81 + F + G4	K2P + I/K3Pu + F/K3Pu + F + G4
ITS + *matK* + *rbcL*	K2P + I/F81 + F/JC + I	K2P + I/K3Pu + F/JC + I
ITS + *psbA‐trnH* + *rbcL*	K2P + I/F81 + F + G4/JC + I	K2P + I/K3Pu + F + G4/TN + F + I
*matK* + *psbA‐trnH* + *rbcL*	F81 + F/HKY + F + G4/JC + I/	K3Pu + F/K3Pu + F + G4/JC + I
ITS+(*matK* + *rbcL*) *+ psbA‐trnH*	K2P + I/(HKY + F + I)/F81 + F + I	K2P + I/(K3Pu + F/JC + I) /K3Pu + F + G4

*Note*: The optimal model of the different molecular markers is indicated on the left and right of the “/” sign.

## APPENDIX 2

**TABLE A2:** Sequences for the phylogenetic tree analysis which are produced in this study.

Organism	Specimen voucher	ITS	*matK*	*psbA‐trnH*	*rbcL*
*Rhododendron* “Taihang Fuxing”	R01	OP922455	OP924120	OP924257	OP924172
*Rhododendron* “Taihang Fuxing”	R02	OP922456	OP924121	OP924258	OP924173
*Rhododendron* “Taihang Fuxing”	R03	OP922457	OP924122	OP924263	OP924174
*Rhododendron* “Taihang Fuxing”	R04	OP922477	OP924123	OP924264	OP924175
*Rhododendron* “Taihang Fuxing”	R05	OP922478	OP924124	OP924265	OP924176
UNNAMED‐1	R06	OP922473	OP924132	OP924224	OP924200
UNNAMED‐1	R07	OP922488	OP924133	OP924230	OP924201
UNNAMED‐1	R08	OP922489	OP924134	OP924231	OP924202
UNNAMED‐1	R09	OP922502	OP924135	OP924239	OP924203
UNNAMED‐1	R10	OP922503	OP924136	OP924240	OP924204
*Rhododendron* “Roseum Elegans”	R11	OP922479	OP924147	OP924241	OP924184
*Rhododendron* “Roseum Elegans”	R12	OP922480	OP924148	OP924242	OP924185
*Rhododendron* “Roseum Elegans”	R13	OP922481	OP924149	OP924243	OP924186
*Rhododendron* “Roseum Elegans”	R14	OP922482	OP924150	OP924244	OP924187
*Rhododendron* “Roseum Elegans”	R15	OP922497	OP924151	OP924245	OP924188
*Rhododendron* “Wilgens Ruby”	R16	OP922466	OP924137	OP924225	OP924205
*Rhododendron* “Wilgens Ruby”	R17	OP922467	OP924138	OP924226	OP924206
*Rhododendron* “Wilgens Ruby”	R18	OP922468	OP924139	OP924232	OP924207
*Rhododendron* “Wilgens Ruby”	R19	OP922483	OP924140	OP924233	OP924208
*Rhododendron* “Wilgens Ruby”	R20	OP922501	OP924141	OP924236	OP924209
*Rhododendron* “Markeetas Prize”	R21	OP922474	OP924142	OP924227	OP924210
*Rhododendron* “Markeetas Prize”	R22	OP922475	OP924143	OP924228	OP924211
*Rhododendron* “Markeetas Prize”	R23	OP922490	OP924144	OP924229	OP924212
*Rhododendron* “Markeetas Prize”	R24	OP922491	OP924145	OP924234	OP924213
*Rhododendron* “Markeetas Prize”	R25	OP922492	OP924146	OP924235	OP924214
*Rhododendron* “Delta”	R26	OP922469	OP924152	OP924247	OP924189
*Rhododendron* “Delta”	R27	OP922470	OP924153	OP924248	OP924190
*Rhododendron* “Delta”	R28	OP922484	OP924154	OP924249	OP924191
*Rhododendron* “Delta”	R29	OP922485	OP924155	OP924250	OP924192
*Rhododendron* “Delta”	R30	OP922486	OP924156	OP924251	OP924193
*Rhododendron* “Rocket”	R31	OP922458	OP924125	OP924259	OP924177
*Rhododendron* “Rocket”	R32	OP922459	OP924126	OP924260	OP924178
*Rhododendron* “Rocket”	R33	OP922460	OP924127	OP924261	OP924179
*Rhododendron* “Rocket”	R34	OP922461	OP924128	OP924262	OP924180
*Rhododendron* “Rocket”	R35	OP922462	OP924129	OP924266	OP924181
UNNAMED‐2	R41	OP922463	OP924160	OP924254	OP924194
UNNAMED‐3	R42	OP922471	OP924157	OP924253	OP924195
UNNAMED‐2	R43	OP922487	OP924161	OP924256	OP924197
UNNAMED‐2	R44	OP922472	OP924162	OP924255	OP924196
UNNAMED‐4	R46	‐	OP924163	OP924270	OP924171
*Rhododendron* calophytum	R51	OP922476	OP924130	OP924271	OP924182
*Rhododendron* calophytum	R52	OP922493	OP924131	OP924272	OP924183
*Rhododendron* ovatum	R56	OP922504	OP924164	OP924269	OP924216
UNNAMED‐5	R61	OP922464	OP924158	OP924246	OP924198
UNNAMED‐5	R62	OP922465	OP924159	OP924252	OP924199
*Rhododendron* delavayi	R66	OP922494	OP924166	OP924222	OP924215
*Rhododendron* delavayi	R67	OP922499	OP924167	OP924223	OP924219
*Rhododendron* “Pink Pearl”	R71	OP922498	OP924169	OP924267	OP924220
*Rhododendron* “Pink Pearl”	R72	OP922500	OP924170	OP924268	OP924221
UNNAMED‐6	R76	OP922495	OP924168	OP924237	OP924217
UNNAMED‐6	R77	OP922496	OP924165	OP924238	OP924218
